# Assessing the impact of adopting a competency-based medical education framework and ACGME-I accreditation on educational outcomes in a family medicine residency program in Abu Dhabi Emirate, United Arab Emirates

**DOI:** 10.3389/fmed.2023.1257213

**Published:** 2024-01-08

**Authors:** Latifa Baynouna AlKetbi, Nico Nagelkerke, Amal A. AlZarouni, Mariam M. AlKuwaiti, Ruwaya AlDhaheri, Amna M. AlNeyadi, Shamma S. AlAlawi, Mouza H. AlKuwaiti

**Affiliations:** ^1^Abu Dhabi Healthcare Services, Ambulatory Healthcare Services, Al Ain, United Arab Emirates; ^2^Community Medicine Department, UAEU, Al Ain, United Arab Emirates

**Keywords:** residency training, milestones, ACGME, in-training exams, competency residency training, performance, competency

## Abstract

**Background:**

Competency-Based Medical Education (CBME) is now mandated by many graduate and undergraduate accreditation standards. Evaluating CBME is essential for quantifying its impact, finding supporting evidence for the efforts invested in accreditation processes, and determining future steps. The Ambulatory Healthcare Services (AHS) family medicine residency program has been accredited by the Accreditation Council of Graduate Medical Education-International (ACGME-I) since 2013. This study aims to report the Abu Dhabi program’s experience in implementing CBME and accreditation.

**Objectives:**

1. Compare the two residents’ cohorts’ performance pre-and post-ACGME-I accreditation.
2. Study the bi-annually reported milestones as a graduating residents’ performance prognostic tool.

**Methods:**

All residents in the program from 2008 to 2019 were included. They are called Cohort one—the intake from 2008 to 2012, before the ACGME accreditation, and Cohort two—the intake from 2013 to 2019, after the ACGME accreditation, with the milestones used. The mandatory annual in-training exam was used as an indication of the change in competency between the two cohorts. Among Cohort two ACGME-I, the biannual milestones data were studied to find the correlation between residents’ early and graduating milestones.

**Results:**

A total of 112 residents were included: 36 in Cohort one and 76 in Cohort two. In Cohort one, before the ACGME accreditation, no significant associations were identified between residents’ graduation in-training exam and their early performance indicators, while in Cohort two, there were significant correlations between almost all performance metrics. Early milestones are correlated with the graduation in-training exam score. Linear regression confirmed this relationship after controlling the residents’ undergraduate Grade Point Average (GPA). Competency development continues to improve even after residents complete training at Post Graduate Year, PGY4, as residents’ achievement in PGY5 continues to improve.

**Conclusion:**

Improved achievement of residents after the introduction of the ACGME-I accreditation is evident. Additionally, the correlation between the graduation in-training exam and graduation milestones, with earlier milestones, suggests a possible use of early milestones in predicting outcomes.

## Introduction

Accreditation in medical education was introduced as an evidence-based process to improve outcomes. Accreditation is defined as the formal evaluation of an educational program, institution, or system, against defined standards, by an external body for quality assurance and enhancement (1). Many reports support that accreditation-related activities steer medical education toward establishing processes that are likely to improve the quality of medical education (2–4). Accrediting bodies increasingly view practice outcome measures as an essential step toward better continuous educational quality improvement (5). Thus, the development of competency-based medical education became an obligatory standard for many accrediting bodies, such as the Accrediting Council of Graduate Medical Education and ACGME-International. The recent introduction of milestones and Entrustable Professional Activities (EPA) is an important development in this area, which needs more research and innovation in its implementation and validation with graduates’ assessment of performance in patient care.

Nevertheless, such an assessment is challenging. While milestones and EPA are works in progress, the milestones completed twice annually have helped programs identify individual residents struggling globally or in a specific area earlier in residency. The process has also helped programs identify areas where the curriculum of the residency program may need improvements (5). On the other hand, competency-based medical education (CBME) implementation creates challenges, such as synchronizing the training of residents, as not all trainees progress at the same speed. Another challenge is that the implementation can be labor-intensive for supervisors and educators.

Implementing CBME may be associated with different challenges in different countries, health systems, or even between PGME and UME in the same country. Therefore, experiences from different programs may not be generalizable, but still, the literature needs evidence in this area. In Abu Dhabi, the government mandated, in 2011, that all residency training should be ACGME-I accredited, and most programs achieved this by 2013. The ACGME-I accreditation signed an agreement with Abu Dhabi Healthcare Services to conduct site visits and accredit programs that meet the ACGME-I standards. The first group of residents for the family medicine residency program of the Ambulatory Healthcare Services (AHS), joined on October 1st, 1994. Since then, more than 160 family physicians have graduated. Its curriculum and process were modified to meet the ACGME-I, which accredited the program in 2013 and renewed the accreditation to date. The milestones were introduced in 2014. There is consistency in the program leadership as the program director, and all core faculty are program graduates who have gone through all or most of the accreditation site visits. In addition, they have attended many ACGME-I faculty development courses conducted over the year, particularly on curriculum and CBME assessments and milestones.

The Family Medicine Program of the Ambulatory Healthcare Services (AHS) was among the first accredited and implemented by all required standards. A vital element of the Accreditation System is the measuring and reporting of educational milestones since 2014.

This study aims to retrospectively reflect on the Abu Dhabi residency program experience in implementing CBME and accreditation. Two cohorts of the AHS Family Medicine program residents were compared pre-and post-ACGME-I accreditation regarding their achievements in different assessments, among them the in-training exam (ITE) scores. Also, the second cohort’s bi-annually reported milestones were used as a prognostic tool for graduating residents’ ITE and milestones.

## Methods

The study is a sectional observational study comparing two cohorts before the ACGME standards implementation and after. Residents included in the study are called Cohort one, whose intake was from 2008 to 2012 before the ACGME accreditation, and Cohort two, whose intake was from 2013 to 2019, after the ACGME accreditation, with the milestones used. Regarding outcome measures, usually, the quality of graduates is best judged by the Board Certification, and in this program, it is the Arab Board examination. The pass rate for the program is above 97% overall, and the results were pass or fail. Therefore, it is not an appropriate outcome for this small cohort. Therefore, it was decided that the in-training exam of the American Board was a better alternative to judge competency achievement. It has the same structure and similar content focus as the Arab Board exam. It is a Multiple-Choice Written exam and covers all current family medicine curricula. Additional competency indicators from the internal program assessments were clinical long-case exams, Objective Structured Clinical Examination (OSCE), oral exams, the yearly in-training exam, and multiple evaluations required by the accrediting body, ACGME-I, which were used as variables in the study of the residents’ competency. Clinical Competency Committees (CCC) review the many different sources of information for each resident at least four times annually and evaluate each resident’s progress across the milestones twice annually. It is worth noting that other than the clinical exams and oral exams, all other assessments were formative and not summative.

All residents in both cohorts have these assessments: yearly clinical exams, oral exams, and yearly OSCE’s and in-training exams. The two cohorts of residents, who were all residents in the program from 2008 to 2019, were compared in all these assessments. The only added assessment in Cohort two, which was not part of Cohort one assessments, was the milestones, which were introduced as part of the required reporting of performance by the accrediting body, the ACGME-I.

Summative assessments included yearly clinical exams and oral exams, while yearly OSCE and in-training exams were obligatory formative assessments. The in-training exam, which is a requirement in the program to be taken by all residents annually, was used as one indication of the change in competency between the two cohorts. In addition, among Cohort two, ACGME-I milestones’ data, which is collected biannually, was studied to find the correlation between the residents’ early and graduating milestones. The program is for four years, but the fifth year in-training exam is available due to extensions for a few or most residents to remain in the program until their certification exam, which is usually during mid-year 5.

Milestones data from residents included 6 or 8 semi-annual reporting periods from July 2016 to June 2019, using the milestones assessment system reported to the ACGME-I. Family medicine is a 3-year ACGME-I accredited training program. However, some residents need to extend training and will have milestones for an extra year. in the first version of the milestones that the program implanted, there were 22 sub-competencies across the 6 ACGME Core Competencies, and now in version 2.0 there are 18: there are five patient care (PC) sub-competencies, two medical knowledge (MK) sub-competencies, two practice-based learning and improvement (PBLI) sub-competencies, three system-based practice (SBP) sub-competencies, two professionalism sub-competencies, and three interpersonal communication skills (ICS) sub-competencies (Table 1).

**Table 1:** The ACGME-I family medicine milestones.

Patient care
PC1 Cares for acutely ill or injured patients in urgent and emergent situations and in all settings
PC2 Cares for patients with chronic conditions
PC3 Partners with the patient, family, and community to improve health through disease prevention and health promotion
PC4 Partners with the patient to address issues of ongoing signs, symptoms, or health concerns that remain over time without clear diagnosis despite evaluation and treatment, in a patient-centered, cost-effective manner
PC5 Performs specialty – appropriate procedures to meet the healthcare needs of individual patients, families, and communities, and is knowledgeable about procedures performed by other specialists to guide their patients’ care
Medical knowledge
MK1 Demonstrates medical knowledge of sufficient breadth and depth to practice family medicine
MK2 Applies critical thinking skills in patient care
System–based practice
SBP1 Patient safety and quality improvement
SBP2 System navigation for patient-centered care
SBP3 Physician role in health care systems
Practice–based learning and improvement
PBLI Evidence-based and informed practice
PBLI Reflective practice and commitment to personal growth
Professionalism
P1 Professional behavior and ethical principles
P2 Accountability/conscientiousness
P3 Self-awareness and help-seeking
Interpersonal and communication skills
ICS1 Patient- and family-centered communication
ICS2 Inter professional and team communication
ICS3 Communication within health care systems

The milestones data are scored between level 1 and level 5 in 0.50-unit intervals (and a preceding level 0 to indicate that the learner has not achieved level 1). Level 4 is specified as the recommended graduation target, which indicates readiness for unsupervised practice. Milestones are designed mainly for formative purposes, including providing feedback on progress made and remaining deficiencies, and are not used for accrediting individual residency programs or eligibility determinations for board certification (6). The value of milestones, and other in-training assessments, depends on the extent to which they measure progress and predict final assessments. A surrogate outcome for board certification was used, namely, the graduates’ in-training exam (at year 4 or 5), which reflects their Board Certification preparedness at the point of completion of their training.

To investigate possible influential factors on outcome in this study, we utilized all other available residents’ data and assessments, including age, gender, medical school GPA, and entry interview score.

Statistical methods used include frequency tables and t-tests. As all outcome variables were continuous variables, correlation coefficients and linear regression analysis were used. A two-tailed significance level of 0.05 was used in statistical tests. For comparing Pearson’s correlation coefficients between the two cohorts, a value of 0.35 was considered significant as it reached the 0.05 significance level in the smaller cohort.

Abu Dhabi Healthcare Services, SEHA, and IRB approved this study. The approval number is SEHA-IRB-035. No consent was needed as the data were retrospectively gathered and anonymized.

## Results

A total of 112 family medicine residents, 36 in Cohort one and 76 in Cohort two, were included. The majority were females (*n* = 34, 94.4% in Cohort one and *n* = 59, 77.6% in Cohort two). The mean ages of graduation for the program were 32.6 years (SD: 3.16) in Cohort one and 28.6 years (SD 2.76) in Cohort two. The GPA average was 2.77 (SD:0.55) in Cohort one and 3.00 (SD: 0.41) in Cohort two. The mean score for the family medicine entry interview was 81.21 (SD: 9.26) in Cohort one and 90.93 (SD:8.86) in Cohort two. All were UAE nationals (Table 2).

**Table 2:** Demographic description of Cohorts one and two of the family medicine residents.

Cohort one					Cohort two				
	Min	Max	Mean	SD	Min	Max	Mean	SD	*p* value
Age	28.0	41.0	32.6	3.2	25.0	43.0	28.6	2.8	<0.001
Entry interview score	57.8	96.9	81.2	9.3	66.0	100.0	90.9	8.9	0.110
GPA	2.0	4.0	2.8	0.6	2.0	3.9	3.0	0.4	0.180
ITE PGY1	200.0	430.0	237.8	52.2	200.0	480.0	242.8	63.1	0.220
ITE PGY2	200.0	420.0	260.0	61.1	200.0	550.0	307.1	95.5	0.530
ITE PGY3	200.0	440.0	285.0	72.2	200.0	560.0	328.5	96.9	0.048
ITE PGY4	200.0	420.0	286.3	66.8	200.0	570.0	343.5	103.8	0.013
ITE PGY5					200.0	690.0	399.6	144.1	
Clinical exam PGY1	4.5	8.0	6.4	0.9	4.5	8.0	6.5	0.8	0.440
clinical exam PGY2	4.0	8.0	6.8	1.0	4.5	8.0	6.5	0.8	0.220
Clinical exam PGY3	4.0	8.0	7.1	1.1	4.0	8.0	6.2	1.0	
Oral exam PGY1	4.0	8.0	6.4	1.1	3.8	8.3	6.2	1.0	0.960
Oral exam PGY2	3.5	8.0	6.4	1.2	1.0	9.0	6.2	1.2	0.960
Oral exam PGY3	1.0	9.0	6.5	1.5	5.0	9.0	6.5	1.1	1.000
OSCE PGY1	37.9	58.8	51.3	6.3	44.2	80.4	62.3	8.5	*p* < 0.0001
OSCE PGY2	49.0	68.0	60.8	6.0	44.2	83.0	67.2	8.6	0.019
OSCE PGY3	59.0	71.0	65.4	4.4	47.0	86.0	69.1	8.8	0.400
OSCE PGY4	51.2	74.9	65.2	7.4	44.6	89.0	73.6	9.6	0.020

**Figure 1:**
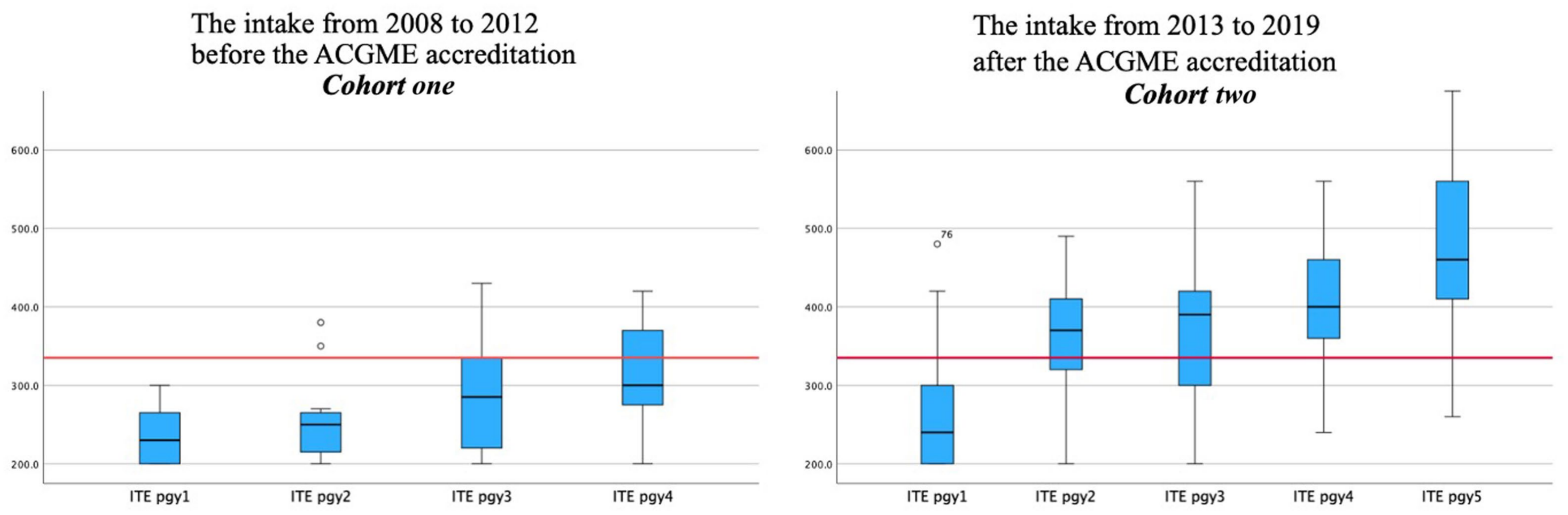
The difference in in-training average score between the two cohorts among PGY1, PGY2, PGY3, PGY4, PGY5.

The mean ITE scores for PGY1 were similar in Cohorts one and two—237.8 in Cohort one, and 242.80 in Cohort two. For PGY2, the mean ITE score was 260 in Cohort one, while it was 307.12 in Cohort two. The mean ITE for PGY3 in Cohort one was 285.04, while in Cohort two, it was 328.54. For PGY4, the mean ITE score was 286.25 in Cohort one, while in Cohort two, it was 343.49. However, there were no significant differences between Cohorts one and two in these ITE scores and other performance, clinical, and oral exams (Table 2; Figure 1).

Studying the correlation between different competency outcomes, using Pearson correlation analysis, found a less significant association between the Cohort one residents’ graduation in-training exam and their early performance in residency years, such as clinical exams. The year one oral exam, year two OSCE, and the in-training exams in years 1 and 2 had significant correlations (*r* = 0.66, *r* = 0.37, *r* = 0.45, *r* = 0.64, respectively) (Table 3). In the newer cohort, Cohort two (2013–2019), there was a more significant correlation between the graduation in-training exam with almost all performance metrics (GPA, OSCE in PGY1 and PGY2, ITE in PGY1 and PGY2, clinical exam in PGY1 and PGY2, oral exam in PGY1 only, and mid and end milestone from in PGY1 to PGY4). More importantly, the graduation in-training exam score was correlated with both ends of the PGY1 milestone (*r* = 0.47), mid-year 2 (*r* = 0.65), and the end of the PGY2 milestone (*r* = 0.71). GPA was not significantly correlated with graduation ITE in both cohorts (Table 3). As shown in Table 4, early, mid, and end year 1 and 2 milestones correlated well with graduation ITE and milestones.

**Table 3:** Pearson correlation of ITE at graduation and variables studied.

**Cohort one**	**ITE PGY4**
Age	0.083
OSCE PGY1	0.192
OSCE PGY2	0.376
ITE PGY 1	0.454*
ITE PGY2	0.649*
ITE PGY 4	1
Sex	0.063
Clinical exam PGY1	0.001
Oral exam PGY 1	0.659*
Oral exam PGY 2	−0.091
Clinical exam PGY2	0.155
GPA	−0.13
**Cohort two**	**ITE pgy4**
Age	0.199
OSCE PGY1	0.363*
OSCE PGY2	0.329*
ITE PGY1	0.550*
ITE PGY2	0.719*
ITE PGY4	1
Mid all milestones average PGY 1	0.321
End all milestones average PGY 1	0.466*
Mid all milestones average PGY 2	0.654*
End all milestones average PGY 2	0.710*
Sex	−0.139
Clinical exam PGY 1	0.293
Oral exam PGY 1	0.453*
Oral exam PGY 2	0.178
Clinical exam PGY 2	0.559*
GPA	0.29

*Significant correlations.

**Table 4:** Correlation between the milestones assessed at different training years for Cohort two which had the milestones implemented.

Pearson’s correlations	Mid all average y1	End all average y1	Mid all average Y2	End all average Y2	MD all average Y3	End all average Y3	MD all average Y4	End all average Y4	ITE y4	ITE y5
Mid all average y1	1	0.480*	0.402*	0.400*	0.255	0.194	0.23	0.375	0.321*	0.646*
End all average y1	0.480*	1	0.699*	0.603*	0.506*	0.501*	0.557*	0.595*	0.466*	0.559*
Mid all average Y2	0.402*	0.699*	1	0.778*	0.598*	0.600*	0.697*	0.798*	0.654*	0.650*
End all average Y2	0.400*	0.603*	0.778*	1	0.775*	0.730*	0.786*	0.713*	0.710*	0.717*
MD all average Y3	0.255	0.506*	0.598*	0.775*	1	0.863*	0.758*	0.670*	0.685*	0.654*
End all average Y3	0.194	0.501*	0.600*	0.730*	0.863*	1	0.858*	0.834*	0.681*	0.637*
MD all average Y4	0.23	0.557*	0.697*	0.786*	0.758*	0.858*	1	0.855*	0.629*	0.641*
End all average Y4	0.375	0.595*	0.798*	0.713*	0.670*	0.834*	0.855*	1	0.680*	0.705*
ITE y4	0.321	0.466*	0.654*	0.710*	0.685*	0.681*	0.629*	0.680*	1	0.841*
ITE y5	0.646*	0.559*	0.650*	0.717*	0.654*	0.637*	0.641*	0.705*	0.841*	1

* Significant correlations.

This relation between early and graduating milestones was confirmed using univariate linear regression except for the very early milestones (mid-first year) (Table 5). With regards to the milestones sub-competencies in patient care, medical knowledge, professionalism, interprofessional and personal skills, system-based practice, and practice-based learning and improvement, linear regression showed a significant prediction of almost all sub-competencies with the ITE in year four after completion of the three ACGME-I programs’ requirements (Table 5). This was for all years’ sub-competencies from PGY1 to PGY3.

**Table 5:** Linear regression of outcome with other early performance variables in Cohort one and Cohort two.

(A) Cohort one: linear regression of graduation IITE as the dependent, PGY4, with other performance variables studied in Cohort one.					
	Unstandardized coefficients		Standardized coefficients	*t*	*p*-value
	B	Std. error	Beta		
OSCE PGY2	3.763	3.094	0.376	1.216	0.255
OSCE PGY 1	2.19	2.999	0.192	0.73	0.477
GPA	−14.309	30.999	−0.127	−0.462	0.652
ITE PGY1	0.947	0.426	0.454	2.224	**0.038**
Clinical exam PGY 1	0.073	16.499	0.001	0.004	0.997
Oral exam PGY 1	38.542	10.682	0.659	3.608	**0.002**
Oral exam PGY 2	−6.262	15.699	−0.091	−0.399	0.694
Clinical exam PGY 2	9.375	12.748	0.155	0.735	0.47

(B) Cohort two: linear regression of PGY4 ITE score as the dependent variable with early (PGY1 – PGY2) milestones sub-competencies and other performance variables in Cohort two.					
	Unstandardized coefficients		Standardized coefficients	*t*	*p*-value
	B	Std. error	Beta		
Individual average of sub-competencies					
Patient care					
Mid patient care PGY1	32.912	30.414	0.167	1.082	0.286
End patient care PGY1	59.359	25.888	0.337	2.293	**0.027**
Mid patient care PGY2	68.726	20.108	0.471	3.418	**0.001**
End patient care PGY2	92.792	22.36	0.544	4.15	**<0.001**
Mid patient care PGY3	101.82	16.713	0.689	6.092	**<0.001**
End patient care PGY3	104.44	21.588	0.603	4.838	**<0.001**
Medical knowledge					
Mid medical knowledge PGY1	47.688	19.094	0.363	2.498	**0.017**
End medical knowledge PGY1	79.86	25.399	0.441	3.144	**0.003**
Mid medical knowledge PGY2	97.87	18.412	0.639	5.316	**<0.001**
End medical knowledge PGY2	81.79	23.979	0.47	3.411	**0.001**
System – based practice					
Mid system – based practice PGY1	56.508	24.321	0.345	2.323	**0.025**
End system – based practice PGY1	51.153	22.869	0.33	2.237	**0.031**
Mid system – based practice PGY2	79.461	23.691	0.464	3.354	**0.002**
End system – based practice PGY2	80.518	22.62	0.486	3.56	**<0.001**
Practice based learning and improvement					
Mid learning and improvement PGY1	57.034	23.803	0.35	2.396	**0.021**
End learning and improvement PGY1	60.755	18.743	0.452	3.241	**0.002**
Mid learning and improvement PGY2	66.268	17.326	0.513	3.825	**<0.001**
End learning and improvement PGY2	80.85	18.52	0.563	4.366	**<0.001**
Professionalism					
Mid professionalism PGY1	13.822	20.431	0.105	0.677	0.503
End professionalism PGY1	23.16	34.368	0.105	0.674	0.504
Mid professionalism PGY2	104.56	32.115	0.453	3.256	**0.002**
End professionalism PGY2	87.359	31.182	0.401	2.802	**0.008**
Interpersonal and communication skills					
Mid interpersonal and communication skills PGY1	20.483	22.076	0.143	0.928	0.359
End interpersonal and communication skills PGY1	−19.909	32.479	−0.095	−0.61	0.543
Mid interpersonal and communication skills PGY2	94.462	28.732	0.457	3.288	**0.002**
End interpersonal and communication skills PGY2	84.921	25.009	0.469	3.396	**0.002**
Overall milestones average of all sub-competencies					
Mid PGY1 average of All	65.673	30.282	0.321	2.169	**0.036**
End PGY1 average of All	132.41	39.243	0.466	3.374	**0.002**
Mid PGY2 average of All	140.57	25.406	0.654	5.533	**<0.001**
End PGY2 average of All	162.42	25.138	0.71	6.461	**<0.001**
Mid PGY3 average of All	133.76	22.214	0.685	6.021	**<0.001**
End PGY3 average of All	146.7	24.655	0.681	5.95	**<0.001**
Exams					
Clinical exam PGY1	35.617	18.143	0.293	1.963	0.056
Oral exam PGY1	54.392	16.705	0.453	3.256	**0.002**
OSCE PGY1	5.351	2.292	0.363	2.335	**0.025**
OSCE PGY2	4.605	2.232	0.329	2.063	**0.047**

Bolded values indicate statistical significance.

**Figure 2:**
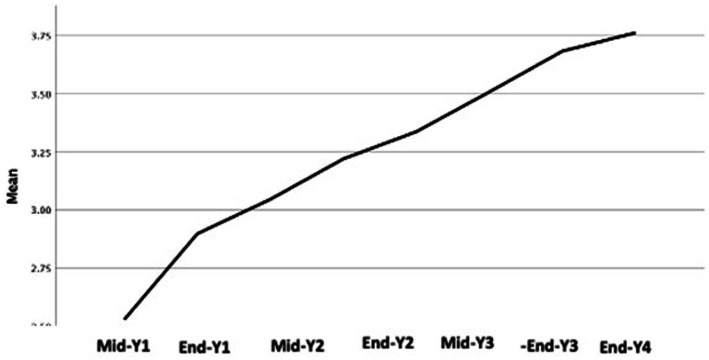
Progress of milestones development over the training years.

It is of value to note that competency development continues with time spent in residency as we can see a continuous development of the residents in the in-training performance and milestones in years 4 and 5. There was no plateau, but the in-training performance and milestones showed continuous improvement (Table 6; Figure 2). A positive correlation exists between graduation ITE score and the early milestone in PGY1 and PGY2. Also, the positive correlation found between GPA and graduation ITE was noted. However, the correlation between graduation GPA and entry interview score was not statistically significant.

**Table 6:** ACGME milestones with the average score of individual competencies and all competencies average distributed by the time in residency training.

**Competency**	**N**	**Min**	**Max**	**Mean**	**S.D.**
Patient care mid PGY 1	76	1	4	2	0.5
Patient care end PGY 1	76	1	4	2.5	0.5
Patient care mid PGY 2	76	1	4	2.7	0.6
Patient care end PGY 2	76	1.3	4	2.9	0.5
Patient care mid PGY 3	75	2	5	3.1	0.7
Patient care end PGY 3	64	2	5	3.5	0.6
Patient care mid PGY 4	52	2	4.6	3.6	0.6
Patient care end PGY 4	21	2.7	4.8	3.7	0.6
Medical knowledge mid PGY 1	76	1	4	2.5	0.7
Medical knowledge end PGY 1	76	1.8	4	2.8	0.5
Medical knowledge mid PGY 2	76	2	4	3	0.6
Medical knowledge end PGY 2	76	2	4.5	3.2	0.6
Medical knowledge mid PGY 3	75	2	4.8	3.4	0.6
Medical knowledge end PGY 3	64	2	5	3.5	0.7
Medical knowledge mid PGY 4	53	2	5	3.8	0.6
Medical knowledge end PGY 4	22	2	5	3.7	0.8
System based practice mid PGY 1	75	1	4	2.1	0.6
System based practice end PGY 1	76	1.1	4	2.4	0.6
System based practice mid PGY 2	76	1.8	4	2.8	0.6
System based practice end PGY 2	76	2	4	3	0.6
System based practice mid PGY 3	75	1.6	5	3.2	0.7
System based practice end PGY 3	64	1.6	4.8	3.4	0.6
System based practice mid PGY 4	51	2	5	3.6	0.7
System based practice end PGY 4	24	0.5	4.7	3.5	0.9
Practice based learning and improvement mid PGY 1	76	1	4	2.3	0.7
Practice based learning and improvement end PGY 1	76	1.8	4	2.8	0.8
Practice based learning and improvement mid PGY 2	76	2	4	3.1	0.8
Practice based learning and improvement end PGY 2	76	1.8	4.3	3.2	0.7
Practice based learning and improvement mid PGY 3	75	2	5	3.5	0.8
Practice based learning and improvement end PGY 3	64	2	4.8	3.6	0.7
Practice based learning and improvement mid PGY 4	51	2	5	3.8	0.8
Practice based learning and improvement end PGY 4	21	2.9	5	4	0.7
Professionalism mid PGY 1	76	1	4	3.4	0.7
Professionalism end PGY 1	76	1.7	4	3.7	0.5
Professionalism mid PGY 2	76	2	5	3.9	0.5
Professionalism end PGY 2	76	2	5	3.9	0.5
Professionalism mid PGY 3	75	2	5	4	0.6
Professionalism end PGY 3	64	2	5	4	0.6
Professionalism mid PGY 4	53	2.7	5	4.1	0.6
Professionalism end PGY 4	20	2.6	5	4	0.7
Interpersonal communication skills mid PGY 1	75	1	4	3.1	0.7
Interpersonal communication skills end PGY 1	75	1.7	4	3.4	0.5
Interpersonal communication skills mid PGY 2	75	2	4	3.5	0.5
Interpersonal communication skills end PGY 2	75	2	4.7	3.6	0.5
Interpersonal communication skills mid PGY 3	74	2.3	5	3.7	0.6
Interpersonal communication skills end PGY 3	63	2.5	5	3.9	0.7
Interpersonal communication skills mid PGY 4	52	2.5	5	4.1	0.6
Interpersonal communication skills end PGY 4	21	2.3	5	4	0.7
**All competency average**	**N**	**Min**	**Max**	**Mean**	**S.D.**
All competency average mid PGY 1	76	1	4	2.6	0.5
All competency average end PGY 1	76	1.7	3.7	2.9	0.4
All competency average mid PGY 2	76	2.2	4.1	3.2	0.4
All competency average end PGY 2	76	2.1	4.2	3.3	0.4
All competency average mid PGY 3	75	2.2	4.6	3.5	0.5
All competency average end PGY 3	64	2.5	4.7	3.7	0.5
All competency average mid PGY 4	54	2.6	4.7	3.8	0.6
All competency average end PGY 4	25	2.5	4.8	3.8	0.6

## Discussion

Abu Dhabi’s investment in medical education, through accreditation, paved the way for better achievement in medical education, and hopefully, this will reflect in a more capable medical workforce. Accreditation system implementation requires restructuring institutions and training programs involved as it implies incorporating quality assurance and outcome tracking systems. The better mean ITE (In Training Examination) score, through academic years PGY1 to PGY4, after the implementation of ACGME accreditation and milestones and the association between the graduating ITE and earlier years’ ITE scores are in line with a systematic review by McCrary et al., which found a moderate-strong relationship between ITE and board examination performance. ITE scores significantly predict board examination scores for most studies. Performing well on an ITE predicts a passing outcome for the board examination, but there is little evidence that performing poorly on an ITE will result in failing in the associated specialty board examination (7).

What explains the improved ITE scores is mainly the implementation of the accreditation standards, as nothing else has changed, and the implementation change is very large. Such standards demanded structure with responsibilities and task distribution within the program. New processes of generating, communicating, and managing performance data were introduced. In addition to adding new curriculum, new teaching initiatives, and the integration of supervised patient care with competency assessments at the workplace. A major change as well is the shift toward competency-based education. The six ACGME competencies were targeted in building curriculum, evaluations, assessments, and target outcomes.

Improving learners’ performance was preplanned based on data fed to the CCC with the generation of milestones bi-annually and the feedback after the data review and actions recommended by the CCC regarding possible residents’ remediation, development opportunities, or promotion decisions. This is a collective decision from multiple sources and expertise and based on gathered observations and data. That was not available before in the case of Cohort one when the Program Director (PD) had to make such performance improvement initiatives and make decisions on learners’ progress. The feedback system to residents through individualized meetings biannually, after milestones assessments, and promotions are required by accreditation standards. Such meetings end with an individualized action plan followed by PD and supervisors for implementation. Weak residents were identified, and an action plan was discussed with them and followed by the PD and CCC committee.

With regards to educational programs, planning of the sessions utilized feedback from the CCC and the PEC and necessitated coverage of all areas of competencies, sometimes, special workshops were added. Curriculum change was a must with the accreditation. For example, there was no structured sports medicine or geriatric services in the city in the time of Cohort one, and no specialist was there in the whole area. When the program was cited for that, a curriculum was built to cover this, and residents had to monitor the volume of elderly patients seen by them, more time was given to both areas with special workshops built to develop the competencies of residents in these areas as sports medicine workshops and in-depth coverage of geriatrics topics.

Additionally, ITE results were utilized to inform the program of better and worse global performance of the residents, which was aimed at in more board review courses and topics in the educational program.

Finally, the milestone is based on ACGME competencies, and among these competencies, as an example, is medical knowledge, which has multiple sources of data. And on a quarterly basis, the CCC will assess progress, and this may influence the In-training performance of residents as it is targeted among other competencies. Additionally, beyond the effect on the ITE score, we provided evidence that these milestones added value to the implemented accreditation system and represented a more comprehensive approach to competency assessment and predicting residency outcomes. Therefore, milestones could be a key player in the difference noticed between Cohorts one and two. It required mandatory tracking and reporting of changes in competency for individual residents of Cohort two. That could not be made without the faculty’s efforts and data collected and analyzed and generating conclusions that were acted upon. Most importantly, the accreditation standards required ensuring structure and processes and a demand for time and resources for the program that supported the residents and faculty, which was not there for Cohort one.

Quantitative and qualitative data for both committees, at different data points from different sources, need to be processed by committee members to be acted upon by Program Director (PD), faculty, and residents (8–10). Assessment tools, developed to assist with milestone assignments, also captured the growth of residents over time and demonstrated quantifiable differences in achievements between PGY classes, which aid timely intervention in supporting residents’ competency development (11). In summary, the difference brought about by the accreditation is a standardized educational structure and processes, with measurements aiming to improve faculty’s accountability in assessment and enhance transparency, and it requires more adherence to administrative tasks and peer assessments from residents and faculty. In a review by Andolsek, starting similar structures and processes as early as in medical schools was recommended to facilitate better resident selection and development (12).

It is worth noting that both GPA and milestones predicted in-training results at graduation. However, while both utilize classic assessment tools like multiple choice questions, MCQs and OSCE, which measure basic medical knowledge, milestones focus on applying acquired knowledge and skills in the workplace assessment and EPA. According to Holmboe and others, milestones data offers a valid and reliable predictive tool that guides the programs to make formal decisions according to the resident’s progress and needs. This might even extend to provide some prediction on the level of the resident’s readiness to face clinical practice (6, 13). Whether this makes milestones data a better predictor of the quality of care that residents provide to patients deserves further empirical study.

Our study showed a positive correlation between the milestone scores and residents’ performance in the graduating written exam; similar findings were noted in other residency programs, such as the Obstetrics and Gynecology and Surgery residency programs, especially in the medical knowledge sub-competency (14–17). The significant prediction of graduation ITE score by the early sub-competencies is an important observation as the ITE is very similar to the exit certifying exam. Therefore, early signs of poor performance, as judged by milestones, can guide targeted remedial actions and provide better support for residents. Although the ACGME-I accredited program is three years long, Arab Board Certification requires four years, and residents can have extensions of their training until mid-year 5, until the Arab Board Certification exam. This provides an opportunity to explore continuous development and performance, especially in years 4 and 5. Residents are given more responsibilities within the practice, and training is more practice-based, which resembles future practice.

Entry interview score in both cohorts, was not significantly linked to any important outcome similar to other reports internationally; a study by Burkhardt et al. did not find consistent, meaningful correlations between the most common selection factors and milestones at any point in the training (18). In this program, the weightage for interview is 75% of the final interview score, and a weight of 25% is given to the GPA. Therefore, we cannot compare our data to other studies as we know that many programs use different entry composite scores that usually include numerical ratings for Dean’s letters and a weighted score for on-site interviews and the GPA. A study by Prystowsky et al. found that higher scores in their program were associated more with the graduate being in an academic faculty position. Therefore, an entry interview alone is difficult to interpret (19). Not surprisingly, the process of selection is challenged and there are increasing calls to reform the US resident selection process as highlighted by a recent scoping review (20). With the use of the milestones initiative, there is a shift from what abilities physicians, in general, should possess to what medical specialists should possess. An increasing number of postgraduate programs have now started to link competencies to Entrustable Professional Activities (EPAs) (21).

Caution in interpreting this study’s findings is essential as there may be alternative explanations for the better performance of Cohort two. For example, more supervisors were program graduates in Cohort two than in Cohort one. Still, as accreditation standards stress investment in faculty development, we can attribute success to accreditation. Finally, these results highlight the value of the competency-based curriculum and assessment metrics during the internship year to better prepare residents for their first residency year and build a handover system between the two graduate educational levels. Ultimately, the ideas underlying the accreditation process should affect the whole medical curriculum.

### Limitation

The outcome, being an exam, does not provide actual work capability in practice. However, the milestones incorporate increasing workplace assessments, which can be a possible strength for milestone assessments. Future research linking milestones to post-graduation performance is crucial to answering this question. And this significant correlation is a start to assessing future performance as graduates. Only one residency program, in one site with two cohorts, was reported in the study, which needs to include a larger number of residency programs.

Our family medicine program is implementing the Entrustable Professional Activities (EPAs) as a further step in competency assessment and development, which will provide future research opportunities to study its impact on the outcome.

## Conclusion

The introduction of the ACGME-I accreditation was associated with increasing the residents’ achievements. Similar to other international studies, milestones proved to be a promising instrument for competency acquisition in the Abu Dhabi AHS family medicine program. The correlation between the graduation in-training exam and graduation milestones with earlier milestones suggests a possible use of early milestones in predicting outcomes.

## Data availability statement

Data is available on request sent to the corresponding author.

## Ethics statement

The studies involving humans were approved by Abu Dhabi Healthcare Services, SEHA IRB. The studies were conducted in accordance with the local legislation and institutional requirements. The ethics committee/institutional review board waived the requirement of written informed consent for participation from the participants or the participants’ legal guardians/next of kin because informed consent was waived by Abu Dhabi Healthcare Services, SEHA, and IRB as the study was designed for retrospective data gathered as part of residency program training and anonymized at analysis.

## Author contributions

LB: Conceptualization, Data curation, Formal analysis, Methodology, Project administration, Writing – original draft. NN: Formal analysis, Writing – review & editing. AAA: Writing – review & editing, MK: Writing – review & editing. RA: Writing – review & editing. AMA: Writing – review & editing. SA: Writing – review & editing. MK: Writing – review & editing.
